# Disseminated fusariosis after allogenic hematopoietic stem cell transplantation: case report

**DOI:** 10.1007/s15010-024-02376-w

**Published:** 2024-08-26

**Authors:** A. Gantner, J. B. Hagemann, B. Grüner, G. Walther, A. Neagoie, V. Wais, H. Döhner, E. Sala

**Affiliations:** 1 Department of Internal Medicine III, University Hospital Ulm, Albert-Einstein-Allee 23, D-89081 Ulm, Germany; 2 Institute of Medical Microbiology and Hygiene, University Hospital of Ulm, Albert-Einstein-Allee 23, D-89081 Ulm, Germany; 3 National Reference Center for Invasive Fungal Infections (NRZMyk), Leibniz Institute of Natural Product Research and Infection Biology–Hans Knöll Institute, Jena, Germany

**Keywords:** Allogenic hematopoietic stem cell transplantation, Fusariosis, Fusarium, Fungal infection

## Abstract

In allogenic stem cell recipients, invasive fungal disease is a common yet dreaded complication with high mortality. Among these, fusariosis is especially complex to treat due to high intrinsic resistance and few antimycotic options, requiring close cooperation of all involved departments. We here report an instructive case of disseminated fusariosis after allogenic stem cell transplantation with fatal outcome despite maximum treatment.

## Case report

A 65-year-old male with a heavily pre-treated high-risk myelofibrosis bearing an ASXL-1 mutation underwent an allogeneic stem cell transplantation (allo-HSCT) from a mismatched (9/10) unrelated donor in 08/2020 after a myeloablative conditioning regimen with Treosulfan 14 g/m² d1-3, Fludarabine 40 mg/m² d1-4 and anti-thymocyte globulin 30 mg/kg. The post-transplant follow-up was complicated due to a poor graft function with continuous transfusion-dependency and grade III-IV neutropenia requiring stimulation with growth factors, which was treated with a stem cell boost. Due to the development of a steroid-refractory acute graft versus host disease (GvHD), the patient underwent further immune suppressive treatment with ruxolitinib during the subsequent follow-up after allo-HSCT.

**Fig. 1:**
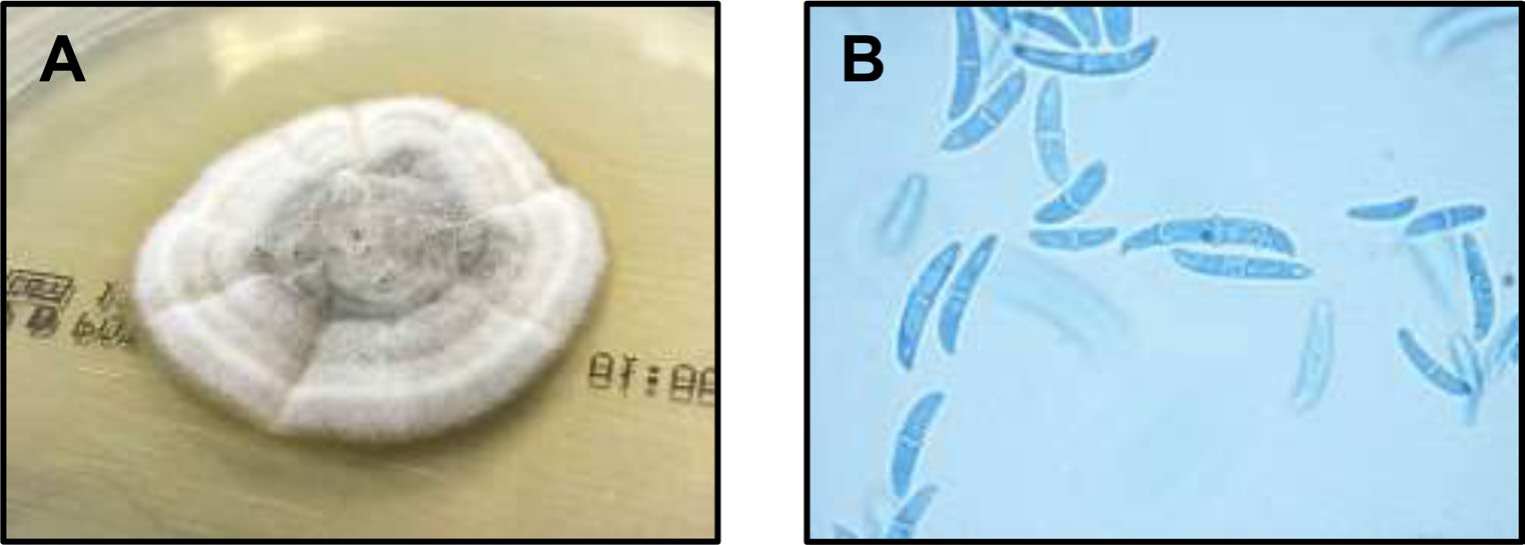
*Fusarium keratoplasticum* isolated from a clinical sample (**A**) Colony growth on Sabouraud-agar after five days of incubation at 30 °C, 5%CO_2_. A cottony surface, darker center and a lighter periphery are common features. (**B**) Lactophenol blue staining with typical large, slightly curved, tapering macroconidia with up to four septa, which are characteristic for *Fusarium* spp

In February 2021, after a blunt trauma of the right foot, the patient presented with a superficial wound next to the toenail of the right hallux. In the performed swabs *Fusarium solani* was detected. Initial treatment was performed with local application of ciclopirox by the patient´s dermatologist and clinical improvement was reported. Four weeks later, the patient developed abdominal pain. Since an ultrasound examination revealed no pathologies, computed tomography (CTI) and magnetic resonance tomography imaging (MRI) were performed, and multiple hepatic lesions suspicious of malignant origin were detected. We admitted the patient to the transplant ward for further diagnostics. During the hospitalization, the patient developed erysipelas on the right foot spreading to the lower leg. Due to persistent neutropenia, continued immunosuppression and eminently elevated CRP levels an empiric antibiotic therapy with meropenem (1000 mg three times per day) and clindamycin (600 mg three times a day) was started. A liver biopsy revealed no malignant cells, but the presence of septate fungal elements. Lactophenol blue staining revealed septate hyphae with branched and unbranched conidiophores. Phialides produced both single-celled, ovoid microconidia and large, slightly curved, tapering macroconidia with up to four septa, which are characteristic for the genus *Fusarium*. The isolate was molecular biologically identified as *F. solani* species complex. Cultural grown *Fusarium* (*F.*) spp. (Fig. 1a, b) were processed for molecular identification. Sequencing of the translation elongation factor 1α (TEF) identified the isolate as *F. keratoplasticum* (Syn. *Neocosmospora keratoplastica)*, a member of the *F. solani* species complex (FSSC). The TEF sequence is stored in GenBank (accession number OR861623) and the corresponding isolate (NRZ-2021-330) is deposited in the Jena Microbial Research Collection (JMRC). Antifungal susceptibility testing by microdilution according to the European Committee on Antimicrobial Susceptibility Testing (EUCAST) protocol revealed the following minimum inhibitory concentrations (MICs): amphotericin B 2 mg/L, isavuconazole > 8 mg/L, itraconazole > 8 mg/L, posaconazole > 8 mg/L, voriconazole > 8 mg/L, anidulafungin > 8 mg/L, and caspofungin > 8 mg/L.

**Fig. 2:**
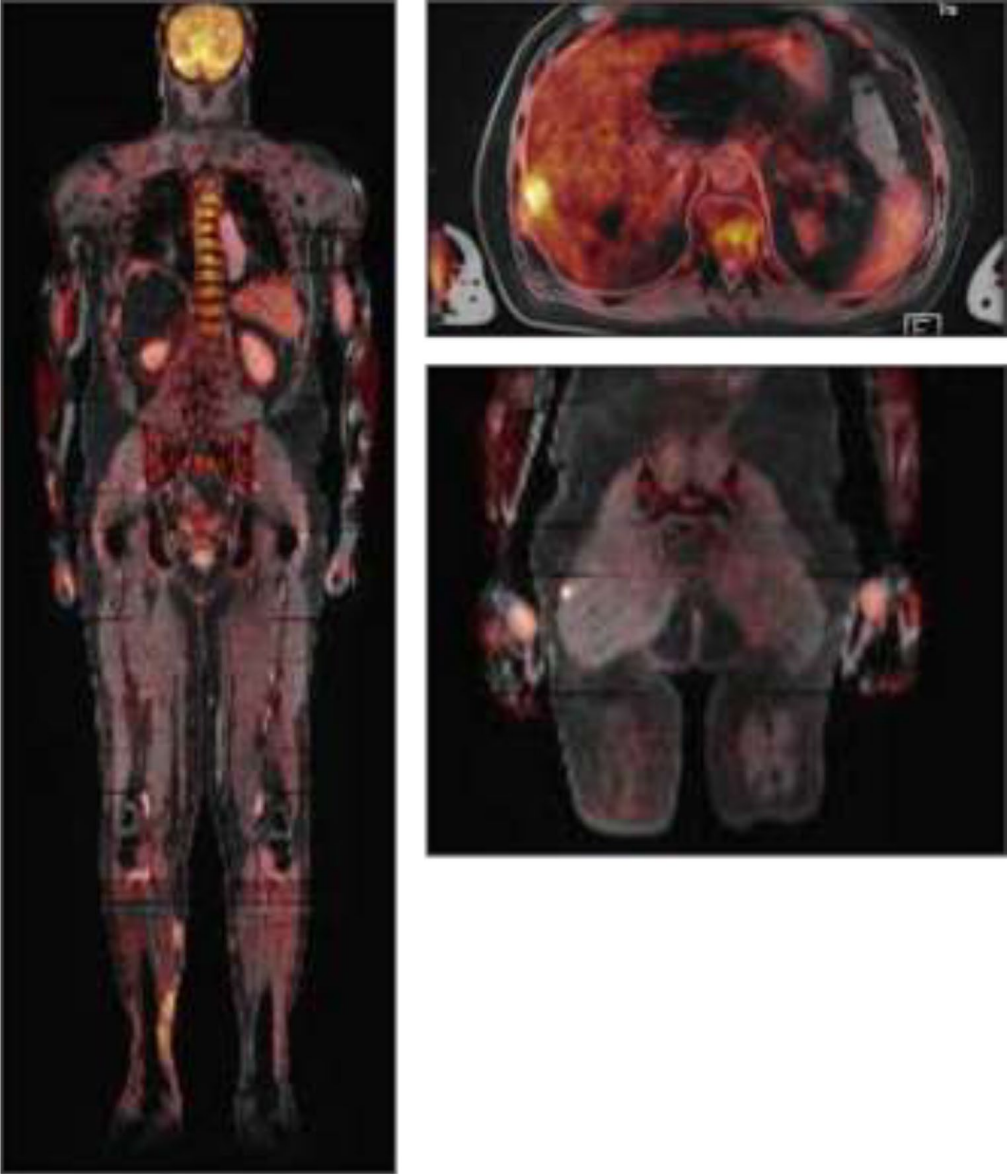
Images of the initial PET-MRI showing lesions with increased metabolism in liver, intramuscular in the glutaeal region and subcutaneous on the medial right lower leg

To clarify the extent of systematic spread of the invasive fusariosis, positron emission tomography (PET)-MRI was performed. Here, additional subcutaneous lesions in the ipsilateral lower leg and multiple intramuscular lesions in the thigh were detected (Fig. 2), retrospectively suggesting fungal dissemination from the initial skin lesion of the right foot.

The patient had received standard antifungal prophylaxis with posaconazole with continuous blood levels within therapeutic range above 1000 µg/l after allo-HSCT, which we switched to a combined intravenous therapy regimen of liposomal amphotericin B (5 mg/kg) plus voriconazole (loading dose 6 mg/kg, 4 mg/kg afterwards). Due to persistent neutropenia with a neutrophil count ranging from 0,2 G/l to 1,2 G/l, granulocyte colony stimulating factor was added. Lymphopenia was also detected, with a lymphocyte count ranging from 0,1 to 0,5 G/l. After a month of intensive antifungal treatment, abdominal CTI showed a stabilization of the hepatic lesions, so surgical resection of the affected liver segments was performed under continued antifungal treatment. Dose adjustment of liposomal amphotericin B to 3 mg/kg was necessary due to progressive renal impairment whereas voriconazole dosage was adjusted to 5 mg/kg on the account of decreased levels in drug monitoring. One month later, in a restaging PET-MRI, a mixed response with residual activity of the subcutaneous lesions and new bipulmonary pleural effusion was observed. Thoracocentesis was performed and *F. keratoplasticum* was subsequently isolated in the pleural effusion as well. According to evidence for other invasive mycoses with pleural affection, local instillation of amphotericin B was initiated under continuation of the systemic antifungal treatment with amphotericin B liposomal and voriconazole [1–3].

At that time, the patient experienced a severe recurrence of the systemic GvHD, so dosage of immunosuppressive treatment, especially steroids, had to be escalated. Under increased immunosuppression, the invasive fusariosis rapidly progressed with worsening of the lung involvemt and persistent cultural evidence of *F. keratoplasticum* in the pleural effusion. Furthermore, bacterial infections with Pseudomonas aeruginosa and Staphylococcus epidermidis emerged, so the patient died 14 weeks after initial diagnosis despite maximal antifungal treatment, due to multiorgan failure caused by uncontrolled bacterial and fungal infection in sustained neutropenia.

## Discussion

Invasive fungal diseases (IFD) are serious complications in allo-HSCT recipients and are associated with high morbidity and mortality. Cumulative incidence lies around 10% [4, 5], and while invasive aspergillosis is the most frequently reported IFD [4], mucormycosis and fusariosis seem to be the second and third most common mould infections, respectively [5–7]. While the incidence of fusariosis is low, mortality is high despite optimal concerted treatment, ranging from 50 to 70% [8, 9].

Cutaneous fusariosis makes up around 70% of cases, particularly in immunocompromised patients, and can either be the primary site of inoculation or a secondary result in systemic dissemination [10]. At the time of clinical presentation, several cutaneous and subcutaneous lesions were observed in our patient, and the likely route of fungal dissemination could be retrospectively reconstructed: *F. keratoplasticum* was first detected in the cutaneous sample of the right hallux, then multiple skin lesions along the right leg, and finally in the liver and in the right pleural cavity were subsequently demonstrated by PET-MRI.

Treatment of invasive fusariosis is challenging. The interpretation of the antifungal susceptibility testing is difficult, mainly due to the absence of break points, to the poor correlation of in-vitro susceptibility data with clinical response, and to multiple intrinsic drug resistances including azoles, echinocandins, and polyenes, which taken together often hamper optimal antifungal therapy [11]. Currently, voriconazole and amphotericin B are considered as first-line antifungal treatment options and combination regimens are also applied [12–15]. Breakthrough fungal infections under posaconazole prophylaxis are reported in around 1–4% of patients, with fusariosis accounting for about 1% of entities in this patient clientele. Whilst lowering the incidence of hitherto more frequent invasive fungal pathogens like *Candida* spp. and *Aspergillus* spp., the broad use of posaconazole as primary antifungal prophylaxis in immunocompromised patients seems to increase the incidence of fusariosis and mucormycosis [6, 7]. Sub-therapeutic posaconazole serum concentration is a major risk factor for infections due to isolates with acquired posaconazole resistance, but breakthrough invasive fungal diseases have also been reported in patients with appropriate serum concentration [6, 16]. Furthermore, numerous *Fusarium* spp. especially of the FSSC are known to have intrinsically high MICs for posaconazole [17]. On the other side, as Nucci and colleagues showed in a relatively large multicentric retrospective study there seems to be no strict correlation between the MIC distribution and the probability of survival of immunocompromised patients developing an invasive fusariosis [18], so that therapeutic decisions should not be at least only based on the MIC profile of a specific Fusarium spp. isolate.

Surgical treatment is considered the therapeutic option of choice whenever possible [12, 13, 15]. In our case, surgical infectious source control and reduction of fungal burden was only possible for the hepatic lesions, while the multiple cutaneous and subcutaneous lesions could not be removed.

Apart from surgical treatment, reconstitution of immunocompetence is considered essential to control fungal infection [12], whereas especially neutropenia is a known risk factor of unsuccessful therapy [7]. In our case the patient had neutropenia and furthermore did not experience T-cell immune reconstitution after transplantation, also considering that termination of immunosuppression was not possible due to severe, steroid-refractory GvHD.

As penetration of systemically administered liposomal amphotericin B into pleural fluid is low [19], local application of non-liposomal amphotericin B can be considered in addition. Local administration of amphotericin B in pleural cavities is usually well tolerated and complete remission of fungal evidence has been reported [1–3].

## Conclusion

IFD remains a major cause of early death after allo-HSCT. While the prevalence of *Candida* spp. infections has decreased after the introduction of azole-based antifungal prophylaxis, there is an increasing number of invasive infections due to *Fusarium* spp. and *Mucorales* [5, 6, 20]. Surgical treatment and improvement of a patient’s immune status are the main backbones for a successful therapy of invasive fusariosis. It has to be considered that surgical treatment could often be difficult due to anatomical peculiarities and/or to the localisation of the fungal infection. On the other side due to the occurrence of acute or chronic GvHD, immunosuppressive treatment is mandatory and impairs the fitness of the immune system, both the innate and the adaptive one. For systemic antifungal treatment of invasive fusariosis, voriconazole and liposomal amphotericin B are currently recommended as substances of choice, and additional local instillation of amphotericin B should be discussed in compartments with restricted bioavailability for its liposomal counterpart, if surgical treatment is not possible. The increasing importance of invasive mycoses leads to an urgent need for new diagnostic and therapeutic options in medical mycology. This is also relevant against the background of global warming, which requires the adaptation of hitherto harmless saprophytic fungi to higher temperatures that could eventually support their survival on or in the human body - underline the urgent need for new diagnostic and therapeutic options in medical mycology. Several novel approaches like olorofim, an inhibitor of the fungal dihydroorotate dehydrogenase, or fosmanogepix are currently under evaluation and might beneficially influence the prognosis of invasive mycoses in the near future [20]. With only few therapeutic options available, uncommon approaches as local instillation should always be evaluated. Still, this case shows that even maximum treatment with combination of antifungal agents, surgery, local instillation and withdrawal of immunosuppression is bound to fail if treatment is started after disseminated and invasive infection in immunocompromised patients. Therefore, awareness needs to be raised in the physicians not used in caring for immunocompromised patient for this rare, but highly aggressive disease, which can present as a harmless cutaneous infection but can reach, especially if not promptly treated, a high mortality rate, ranging from 50 to 70% of affected patients.

## Data Availability

No datasets were generated or analysed during the current study.
